# Possible Compensatory Role of ASICs in Glutamatergic Synapses

**DOI:** 10.3390/ijms241612974

**Published:** 2023-08-19

**Authors:** Konstantin K. Evlanenkov, Arseniy S. Zhigulin, Denis B. Tikhonov

**Affiliations:** Sechenov Institute of Evolutionary Physiology and Biochemistry RAS, St. Petersburg 194223, Russia; konstantin361@mail.ru (K.K.E.); arseniy.zhigulin@yandex.ru (A.S.Z.)

**Keywords:** synaptic transmission, acidification, postsynaptic receptors, inhibition, activation

## Abstract

Proton-gated channels of the ASIC family are widely distributed in central neurons, suggesting their role in common neurophysiological functions. They are involved in glutamatergic neurotransmission and synaptic plasticity; however, the exact function of these channels remains unclear. One problem is that acidification of the synaptic cleft due to the acidic content of synaptic vesicles has opposite effects on ionotropic glutamate receptors and ASICs. Thus, the pH values required to activate ASICs strongly inhibit AMPA receptors and almost completely inhibit NMDA receptors. This, in turn, suggests that ASICs can provide compensation for post-synaptic responses in the case of significant acidifications. We tested this hypothesis by patch-clamp recordings of rat brain neuron responses to acidifications and glutamate receptor agonists at different pH values. Hippocampal pyramidal neurons have much lower ASICs than glutamate receptor responses, whereas striatal interneurons show the opposite ratio. Cortical pyramidal neurons and hippocampal interneurons show similar amplitudes in their responses to acidification and glutamate. Consequently, the total response to glutamate agonists at different pH levels remains rather stable up to pH 6.2. Besides these pH effects, the relationship between the responses mediated by glutamate receptors and ASICs depends on the presence of Mg^2+^ and the membrane voltage. Together, these factors create a complex picture that provides a framework for understanding the role of ASICs in synaptic transmission and synaptic plasticity.

## 1. Introduction

Proton-gated channels of the acid-sensitive ion channel (ASIC) family are widely distributed in central neurons and are found in the cortex, cerebellum, amygdala, hippocampus, and many other structures [1,2]. Both principal glutamatergic neurons and inhibitory interneurons express ASICs [3]; however, ASICs in glial cells are less well-studied, despite their potentially important function in glia [4]. This distribution of ASICs suggests that these structures participate in some basic neurophysiological processes, in addition to providing pH sensitivity to some specific cell types. Among the ASIC subunits, only ASIC3 provides a significant steady-state response to acidification [5], but it is poorly expressed in central neurons. Although ASIC2a homomeric channels also demonstrate a steady-state response to acidification, neuronal ASICs, which consist of ASIC1(a/b) and ASIC2(a/b) subunits, are completely desensitized in a few seconds; therefore, they cannot respond to slow changes in extracellular pH [6,7]. Even weak acidifications, which do not evoke any significant response, cause steady-state desensitization that makes the ASICs insensitive to further significant acidifications [8]. These biophysical characteristics of neuronal ASICs imply that they should be involved in some fast processes that are accompanied by fast transient acidifications.

Fast acidifications take place during synaptic transmission, as the accumulation of neurotransmitters in synaptic vesicles includes proton pumping, which results in acidic content. During synaptic release, the protons fuse in the cleft and can activate postsynaptic ASICs simultaneously with the activation of other post-synaptic receptors. Indeed, ASIC-mediated components have been found in several synapses [9,10,11,12,13]. Since ASICs are sodium-selective, their activation contributes to postsynaptic depolarization. However, ASIC-mediated currents in glutamatergic synapses are subtle, and specific measures are required to register them and prove their origin [1]. This apparently conflicts with the data on ASIC responses to applied acidic solutions, as the response amplitudes are comparable to the responses to glutamate and its agonists. The synaptic acidifications observed in experiments in vitro, which use pH buffers, are likely insufficient to cause significant activation of ASICs [9,10,13]. Indeed, the use of agents that increase ASIC sensitivity to protons increases the ASIC-mediated components of transmission [14]. This suggests that, under physiological conditions, the synaptic acidifications are sufficiently strong to cause significant ASIC responses. Nevertheless, an important consideration is that an increase in the proton concentration in the synaptic cleft not only activates ASICs, but also inhibits NMDA- and AMPA-type ionotropic glutamate receptors. 

The extracellular protons target many important proteins, including the ion channels. For instance, sodium channels [15,16,17] and nicotinic cholinoreceptors [18,19] are inhibited by protons. Extracellular acidification results in inhibition of the ionotropic glutamate receptors. The pH_50_ for AMPA receptor inhibition is about pH 6.2 [20,21]. The NMDA receptors are even more sensitive; some NMDA receptors are inhibited even at neutral pH [22]. By comparison, the pH_50_ values for neuronal ASIC activation are usually between pH 6.6 and 6.0, meaning that no pH conditions can provide ASIC activation without inhibiting NMDA and AMPA receptors. The opposite effects of protons on ASICs and ionotropic glutamate receptors suggest a possible role of ASICs in neural transmission. In particular, the inhibition of glutamate receptors by extracellular protons could be compensated by ASIC activation. This effect could provide stability of the total response under different acidification conditions.

Regrettably, the precise measurement of synaptic acidification is not possible. Therefore, we have tested the ability of ASICs to compensate for glutamate receptor inhibition by performing in vitro experiments using the patch-clamp technique on isolated rat brain neurons. We found that, for some types of neurons, the relationship between ASICs and glutamate receptors and their pH dependencies does provide stability of total response to glutamate and its agonists at different pH values, although both components are strongly pH-dependent.

## 2. Results

We initially characterized the ASIC and glutamate receptor responses in several types of neurons, which were isolated from the brain slices and identified by their morphological and physiological characteristics. Pyramidal neurons from the CA1 area of the hippocampus have a characteristic shape, and their AMPA receptors are calcium-impermeable and can be tested using IEM-1460, a selective channel-blocker of calcium-permeable AMPA receptors [23]. The same is true for the pyramidal neurons of the cortex. The non-pyramidal neurons (interneurons) of the hippocampus express calcium-permeable AMPA receptors that are highly sensitive to IEM-1460 [24,25]. The interneurons of the striatum are large in size and their AMPA receptors are also calcium-permeable [24,25]. We characterized the ASIC responses of these neurons in our previous papers [6,26,27,28,29], in particular, by using specific pharmacological agents. In the present study, we performed a combined analysis of ASICs and glutamate receptors.

**Figure 1:**
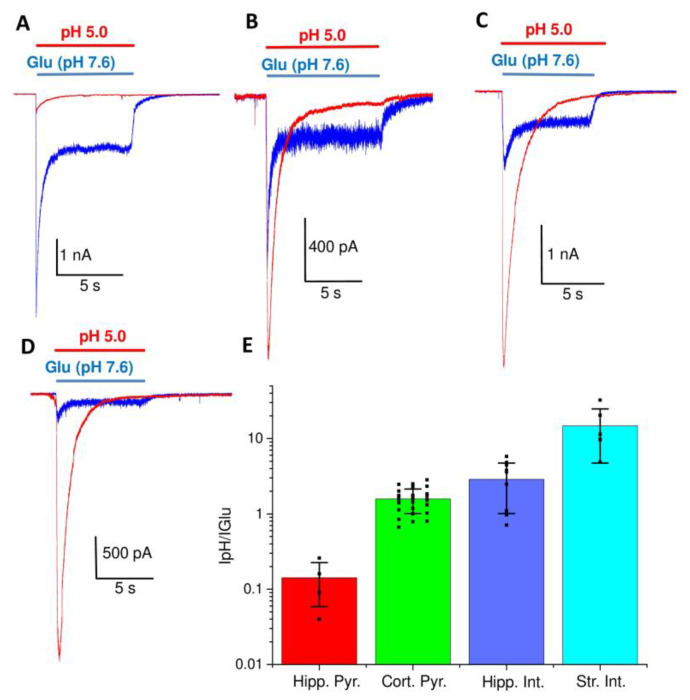
Comparison of responses of isolated rat brain neurons to glutamate at pH 7.6 and to acidification. (**A**–**D**), representative recordings from pyramidal neurons of the hippocampal CA1 area (**A**), interneurons from the hippocampus CA1 area (**B**), pyramidal neuron of the prefrontal cortex (**C**), and giant interneurons of the striatum (**D**). Responses of glutamate receptors (blue) were evoked by application of 3 mM glutamate 10 µM Gly. ASIC responses (red) were evoked by pH drop to 5.0. The colors of the application bars correspond to the color of the current recording. Note, the amplitude scale bars are different for (**A**–**D**). (**E**), ratio of the peak current evoked by acidification to pH 5.0 (IpH), and to glutamate at pH 7.6 (IGlu).

We compared the responses of glutamate receptors and ASICs using saturating concentrations of glutamate (3 mM Glu plus 10 µM Gly) and pH 5.0, respectively. Although pH 5.0 is not a saturating acidification, especially for ASIC2a channels, this value corresponds to almost complete inhibition of glutamate receptors. Possible ASIC activation at more acidic pH values is not essential for the purposes of our study. The results are shown in Figure 1. The pyramidal neurons of the hippocampus produced large (3–12 nA) responses to glutamate, and only subtle (0.1–0.5 nA) responses to acidification (Figure 1A). By contrast, the responses by the giant striatal interneurons to pH 5.0 (3–10 nA) were much larger than their responses to glutamate (~300 pA) (Figure 1D). An intermediate situation was seen in hippocampal interneurons and cortical pyramidal neurons (Figure 1B,C), in which the responses to glutamate and low pH were comparable in amplitude. These results are interesting per se because they show a non-uniform distribution of ASICs and glutamate receptors in different types of neurons. This distribution does not correlate with primary function, as the two types of pyramidal neurons differ drastically, as do the two types of interneurons. The specificity of expression should reflect a more delicate tuning of their properties. Another non-trivial result is that, in many neurons, the ASICs (whose functional role is believed to be modest) are capable of generating currents that are comparable to, or even larger than, the responses to glutamate, which mediates the main excitatory synaptic input.

The proposed compensatory role of ASICs is hardly possible for hippocampal pyramidal neurons and striatal interneurons because the responses to glutamate and low pH differ so drastically (Figure 1E). In the hippocampal interneurons and cortical pyramidal neurons, compensation is possible and can be studied in detail. However, the interneurons of the hippocampus form a heterogeneous group; therefore, we decided to continue our study using cortical pyramidal neurons. 

**Figure 2:**
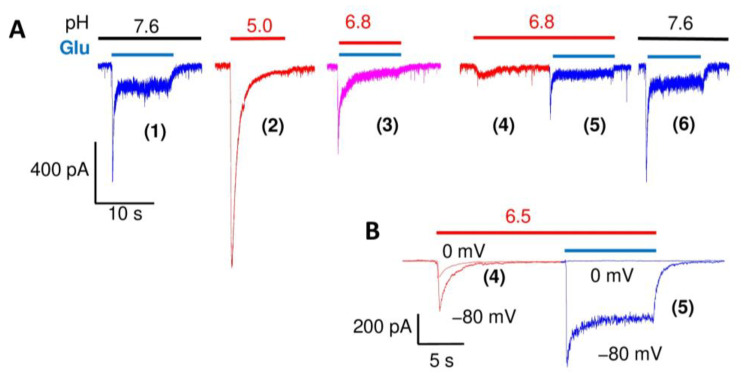
(**A**), representative recordings illustrating the experimental protocol used for analysis of the responses of glutamate receptors and ASICs at different pH values (pH 6.8 is shown). Numbers in brackets indicate: (1), control response to glutamate; (2), control response to pH drop; (3), total response to testing acidification (pH 6.8) and glutamate; (4), response to testing acidification in the absence of glutamate; (5), response to glutamate after acidification; (6), check for glutamate response stability. Responses to glutamate, acidification, and simultaneous application of glutamate and low pH are shown in blue, red, and magenta, respectively. See text for details. (**B**), control of the nature of responses 4 and 5 at 0 mV. ASIC-mediated current (4) remains inward; the response to subsequent glutamate application disappears at 0 mV.

The experimental protocol is shown in Figure 2A. We initially recorded the control responses to glutamate at pH 7.6 (response 1 in Figure 2A) and the response to pH 5.0 in the absence of glutamate (response 2 in Figure 2A). We then applied glutamate simultaneously with acidification to a specific pH value (pH 6.8, response 3 in Figure 2A) to simulate the synaptic conditions and obtain the total response. We separated this response into the components mediated by glutamate receptors and ASICs by first applying a low pH in the absence of glutamate (response 4) to record the ASIC-mediated component and then adding glutamate without restoring a neutral pH (response 5). Since the native ASICs desensitized completely, this application of glutamate activated only glutamate receptors at pH 6.8. In other words, this recording shows how the glutamate receptors are affected by different acidic conditions. Finally, the application of glutamate at pH 7.6 and acidification to pH 5.0 were repeated (only the response to glutamate (6) is shown in Figure 2A). This was necessary to control the recording stability. If the currents at the end of the experiment differed from initial responses by more than 10%, the experimental data were excluded from further analyses. Since the submaximal activation of ASICs induced by the mild proton concentration used, the inactivation could be only partial. To ensure the nature of separated responses 4 and 5, we recorded the currents at 0 mV. Non-selective cation currents mediated by glutamate receptors should reverse at 0 mV, while reversal voltage for sodium selective ASIC-mediated currents is positive. Figure 2B demonstrates the results. Indeed, at 0 mV, we can see a well-detectable response to pH drops to 6.5, whereas response to a subsequent application of glutamate did not cause any response. Thus, responses 4 and 5 in Figure 2 represent ASIC-mediated currents (response 4) and glutamate-gated currents (response 5).

**Figure 3:**
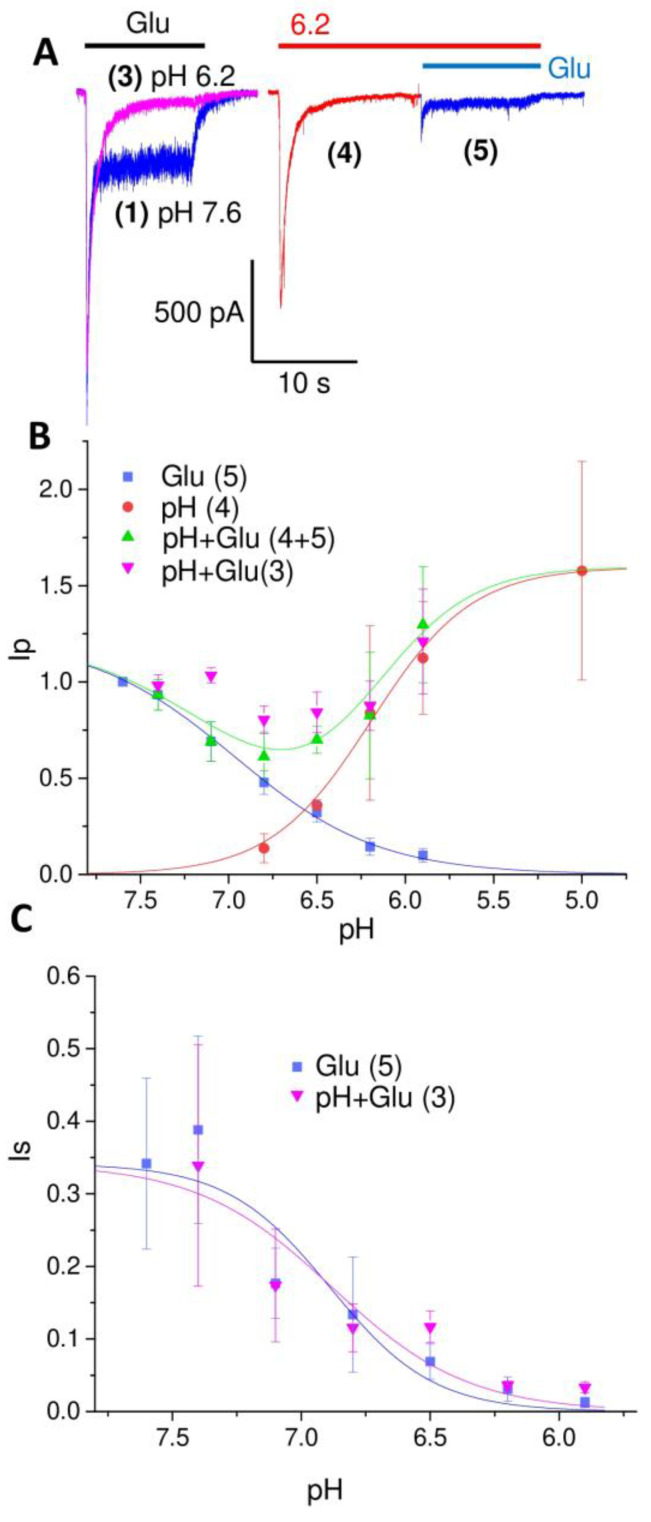
Responses of the glutamate receptors and ASICs at different pH values in the pyramidal neurons of the prefrontal cortex. (**A**), representative recordings at pH 6.2. Numbers in brackets specify responses in the experimental protocol (Figure 2). Responses to glutamate, acidification, and simultaneous application of glutamate and low pH are shown in blue, red, and magenta, respectively. The peak response to glutamate at pH 6.2 (magenta) is only slightly smaller than the control response at pH 7.6 (blue). This is due to the activation of ASICs (red) that compensate for the inhibition of glutamate receptors by protons (blue). (**B**,**C**), pH-dependence of the peak (Ip, (**B**)) and sustained (Is, (**C**)) components of the responses. Green symbols (pH + Glu (4 + 5)) show the sum of the components mediated by ASICs and glutamate receptors. Magenta symbols (Ph + Glu (3)) show the currents evoked by simultaneous application of glutamate and low pH. In panels (**B**,**C**), the Ip and Is are dimensionless values obtained by normalizing to the response to glutamate at pH 7.6. Smooth curves were obtained by fitting the experimental data by Hill equation (see Section 4).

The low pH of pH 6.8 in Figure 2 was varied from 7.4 to 5.9 (Figure 3). Since the currents at pH 6.8 are already exemplified in Figure 2, Figure 3A shows the currents at pH 6.2. The peak (Ip) and steady-state components (Is) of the glutamate response (response 5) decreased with acidification. The pH_50_ value for the proton inhibition of the peak component of the response to glutamate was 6.97 (IC_50_ = 0.11 ± 0.02 µM). This value is close to the proton inhibition value for NMDA receptors [22]. The ASIC-mediated currents (response 4) increased with pH_50_ 6.19 (EC_50_ = 0.64 ± 0.13 µM) and showed a maximal response of 1.6 ± 0.11. As a result of this combination, the peak component of the total response (response 3) demonstrated only a small decrease (20 ± 7%, n = 5 at pH 6.8) at pH values between 7.0 and 6.0. At pH 6.5, the ASIC and glutamate receptors produced almost equal contributions to the total response. At more acidic pH values, the total response exceeded the glutamate response at pH 7.6. The sustained component of the complex response (3) decreased with pH_50_ 6.89 (IC_50_ = 0.13 ± 0.03 µM). Since the ASIC-mediated currents desensitized completely, the total steady state response (3) matched the steady-state response to glutamate (5) and demonstrated the same pH-dependence with pH_50_ 6.88 (IC_50_ = 0.13 ± 0.02 µM). Note that the peak and sustained components of the response to glutamate were equally inhibited by protons. 

**Figure 4:**
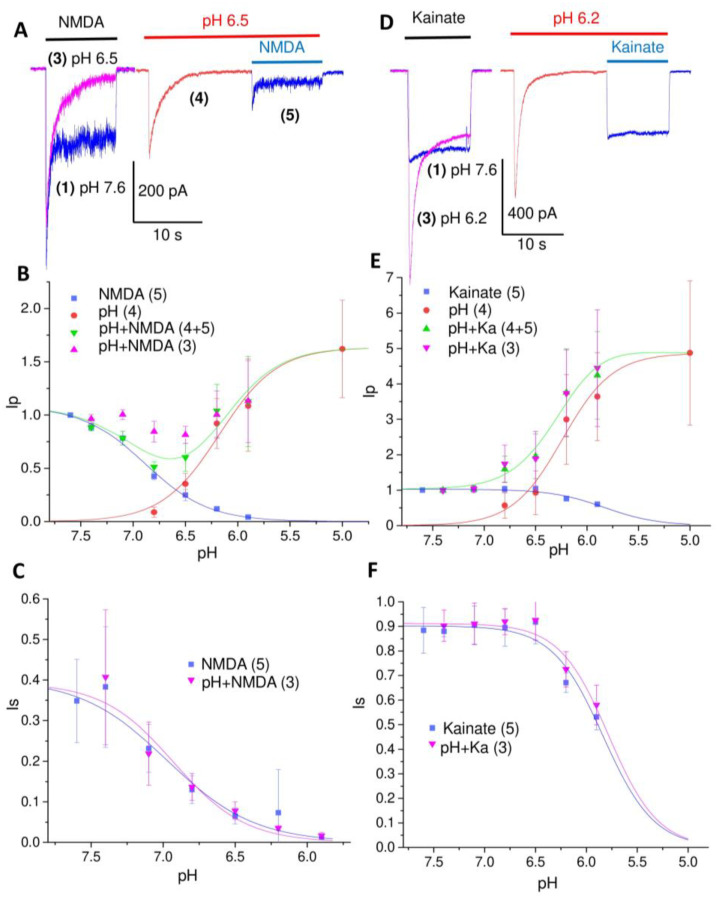
Responses of glutamate receptors and ASICs at different pH values to NMDA (**A**–**C**) and kainate (**D**–**F**) in pyramidal neurons of the prefrontal cortex. (**A**,**D**), representative recordings for pH 6.5 (**A**) and 6.2 (**D**). Numbers in brackets specify responses in the experimental protocol (Figure 2). Responses to glutamate agonists, acidification, and simultaneous application of glutamate and low pH are shown in blue, red, and magenta, respectively. (**A**), the response to NMDA at low pH (3) is only slightly smaller than the control response at pH 7.6 (1). This is due to the activation of ASICs (4) that compensates for the inhibition of NMDA receptors by protons (5). (**D**), Responses to kainate are weakly inhibited by protons. As a result, the response to kainate at low pH is increased due to the activation of ASICs. (**B**,**C**,**E**,**F**) pH-dependence of peak (Ip, (**B**,**E**)) and steady-state (Is, (**C**,**F**)) components of the responses. Green symbols (pH + NMDA or kainate) show the sum of the components mediated by ASICs (response 4) and glutamate receptors (response 5). Magenta symbols (pH + NMDA or kainate (3)) show the currents evoked by simultaneous application of GluR agonist and low pH. In panels (**B**,**C**,**E**,**F**), the Ip and Is are dimensionless values obtained by normalizing to the response to NMDA or kainate at pH 7.6. Smooth curves were obtained by fitting the experimental data by Hill equation (see Section 4).

The responses of neurons to glutamate included the parts mediated by NMDA and AMPA receptors. Additional analysis was performed with the same protocol, but used NMDA (300 µM plus 10 µM Gly) and kainate (1 mM) for selective activation of these types of glutamate receptors. The results are shown in Figure 4. Panels A and D show the representative currents at pH 6.5 (A), which are not exemplified above, and at pH 6.2 (D), where response to kainate was only slightly lower than that at pH 7.6. The steady-state responses to NMDA demonstrated a pH_50_ of 6.97 (IC_50_ = 0.11 ± 0.04 µM) and 6.93 for the total response (0.12 ± 0.03 µM). The total response demonstrated only a minor (19 ± 8%, n = 6) decrease at pH 6.5 compared to the control response at pH 7.6. This result agrees with the ratio of the control NMDA receptor response at pH 7.6 and the ASIC response at pH 5.0 (1.62 ± 0.46, n = 18), which is similar to the ratio of the total response to glutamate and to pH 5.0. Thus, the ASIC-mediated compensation of the NMDA receptor responses agreed well with the effects on the total responses to glutamate. The steady-state responses to kainate at pH 7.6 were much smaller than the responses to acidification at pH 5.0 (4.9 ± 2.0, n = 15). As a result, no compensation effect was seen, and the total response increased monotonically with acidification. Inhibition of the kainate response demonstrated a pH_50_ of 5.83 (IC_50_ = 1.47 ± 0.13 µM) for the peak and 5.78 (IC_50_ = 1.66 ± 0.2 µM) for the total response.

**Figure 5:**
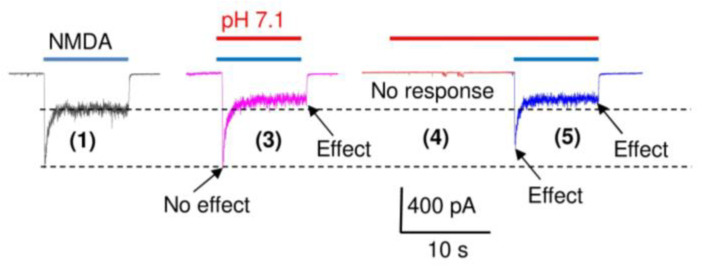
Possible cause of disagreement between the total response (response 3 in Figure 2) and the sum of responses to pH drop (response 4) and to NMDA (response 5). Responses to NMDA, acidification, and simultaneous application of NMDA and low pH are shown in blue, red, and magenta, respectively. Inhibitory effect of weak acidification (pH 7.1) develops slowly. As a result, the peak current of response 3 is unaffected, while the steady-state current is inhibited. If the acidification is applied before NMDA receptor activation, both peak and steady-state components are equally affected (response 5). Note the absence of response to pH drop (response 4), suggesting the entire response 3 is mediated by NMDA receptors.

Figure 3B and Figure 4B demonstrate an interesting peculiarity. The sum of the amplitudes of the responses to acidification (4) and to glutamate (5) (Figure 3B) or NMDA (Figure 4B), which are shown in green, are smaller than the amplitude of the total response (3, shown in magenta). One possible explanation for this effect is the recently found potentiating effect of glutamate on ASIC1a [30]. We checked this possibility by activating ASICs by pH drops to 6.5, both in the presence and in the absence of 3 mM glutamate. The glutamate receptors were inhibited by a combination of DNQX (10 µM), GYKI (200 µM), Mg^2+^ (5 mM), and the absence of Gly. This composition of antagonists eliminates the response to glutamate almost completely. However, the responses to acidification, both in the absence and in the presence of glutamate, did not differ significantly. Glutamate, as well as other selective ASIC1a potentiators such as histamine [31], are unlikely to potentiate native heteromeric ASICs [28]. An alternative explanation is that extracellular protons cause stronger inhibition of the peak NMDA receptor response if they are applied prior to the receptor activation (response 5), as compared with application simultaneously with activation (response 3). Indeed, Figure 4B,C and Figure 5 (representative recordings at pH 7.1) demonstrate that, at pH 7.1, no significant ASIC-mediated currents (response 4) are seen, and response 3 (simultaneous application of protons and NMDA) is mediated by NMDA receptors only. The peak component of this response is not affected significantly (0 ± 5%, n = 5), whereas the steady-state response is inhibited by 28 ± 5% (n = 5). At this pH value, both peak and steady-state components of response 5 (protons are applied before the receptor activation) are inhibited equally at 22 ± 7% (n = 5) and 26 ± 3% (n = 5), correspondingly. Likely, the kinetics of proton block is not fast enough to provide full development of the effect on the peak current if the protons are applied simultaneously with activation.

The data on NMDA and kainate do not allow a straightforward Interpretation, because their amplitudes do not necessarily correlate with the relative contributions of NMDA and AMPA receptors in the total response to glutamate. The action of these agonists differs from the action of glutamate by the channel opening probability and desensitization. The important role of NMDA receptors raises a question of the NMDA receptor contribution in the presence of physiological concentrations of magnesium ion, which blocks NMDA receptor channels near the resting potential [32]. Magnesium also affects ASICs [8]. Therefore, we performed another series of experiments in which the channels were activated by glutamate in the presence of 1 mM Mg^2+^.

**Figure 6:**
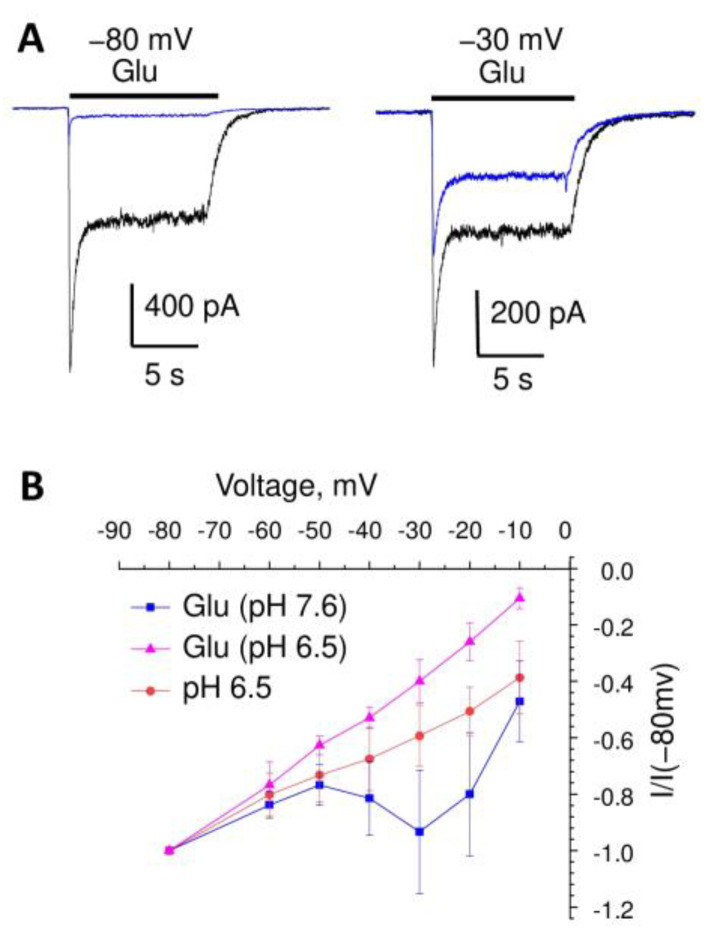
Effects of a voltage-dependent magnesium block of NMDA receptors on the glutamate responses at different pH values. (**A**), representative recordings of the responses to glutamate in control (black) and in the presence of 1 mM Mg^2+^ (blue) at −80 and −30 mV. Due to the voltage-dependence of Mg^2+^ action, the inhibition is much smaller at −30 mV than at −80 mV. (**B**), voltage-dependence of responses to glutamate at pH 7.6 (blue) and 6.5 (magenta) in the presence of 1 mM Mg^2+^ and at a pH drop to 6.5 (red). The I/V plot for pH 7.6 is biphasic due to the voltage-dependent block of the NMDA receptors. At pH 6.5, the plot is almost linear, since the NMDA receptors are inhibited by protons. Due to the sodium selectivity of the ASICs, their responses reverse at positive voltages. As a result, the fraction mediated by ASICs increases with depolarization.

At pH 7.6, Mg^2+^ caused 80 ± 5% (n = 5) inhibition of the response to glutamate, thereby confirming that the dominant fraction of the response is mediated by NMDA receptors (Figure 6A). At −30 mV, the application of 1 mM Mg^2+^ produced an incomplete block of the NMDA receptors, while the total response to glutamate was inhibited by 56 ± 9%(n = 5) only (Figure 6A). As a result, the I/V plot for the glutamate response in the presence of magnesium (Figure 6B) is complex. The linear part from −80 to −60 mV is mediated mainly by AMPA receptors, whereas the increased response around −30 mV reflects the contribution of NMDA receptors. Both components decrease to a zero value around 0 mV. At pH 6.5, which strongly inhibits the NMDA component, the I/V plot was almost linear. In agreement with the sodium selectivity of ASICs, the response to a pH drop from 7.6 to 6.5 was linear, but was reversed at positive voltages. Notably, the ASIC contribution to the total response became more prominent with membrane depolarization.

## 3. Discussion

In the present study, we characterized the responses of rat brain neurons to external acidification and glutamate receptor agonists (glutamate, NMDA, kainate). Our results reproduced previously known effects of ASIC activation and inhibition of glutamate receptors by protons. The total response to glutamate at different proton concentrations is the sum of these independent non-interfering components mediated by glutamate receptors and ASICs. This total response was rather stable over the wide range of extracellular pH. Thus, activation of ASICs can compensate for the inhibition of NMDA and AMPA receptors by protons in the case of synaptic cleft acidification during glutamate release. This mechanism can explain the important role of ASICs in synaptic processes, even though the ASIC-mediated currents are subtle in experiments in vitro, which necessarily use pH buffers. The effect of buffer concentration on ASIC-mediated responses during synaptic transmission has been studied previously [9,10,13]. Lowering the buffer capacity enhances the ASIC-mediated currents because the buffer prevents the synaptic cleft acidification. However, the second probable component of the effect of buffer capacity, namely the influence of protons on the response mediated by glutamate receptors, remains poorly studied. Interestingly, buffer capacity affects both components of the postsynaptic response and its total amplitude. The total postsynaptic response, which includes components mediated by ASICs and glutamate receptors, should depend on the ratio between the receptors, as well as on the ratio between released protons and glutamate molecules.

These relationships are very complex because they involve both pre- and post-synaptic components that, in turn, are controlled by numerous factors. Little is known about the realistic ratio of protons and glutamate molecules in the synaptic cleft and on the factors that can control this ratio. The proton gradient that drives neurotransmitter loading is formed by vacuolar adenosine triphosphatases, and both the pump and exchanger can be regulated [33,34] during various processes. Although the pH in synaptic vesicles is about 5.5 [35], it is hardly possible that such acidification takes place in the synaptic cleft, because ionotropic glutamate receptors are almost completely inhibited at these conditions. We can hypothesize, at the level of speculation, that the ratio between protons and glutamate molecules at the postsynaptic membrane is dynamic and can be significantly different under conditions which are associated with synaptic plasticity and (or) pathological processes. If so, inhibition of glutamate receptors by protons and activation of ASICs, which could compensate this effect, is expected to modulate synaptic transmission.

The voltage-dependent block of NMDA receptors by magnesium ions adds an extra complication to the picture. Besides magnesium, other endogenous compounds can have complex effects on the transmission. For instance, spermine potentiates ASICs [8,36] and their synaptic currents [14]. Another ASIC potentiator is histamine [14,31]. Both these compounds also affect glutamate receptors [37]. Unfortunately, their effects on the two components of glutamatergic transmission have usually been studied separately, so the total effects remain unclear. The complexity of the relationships between the responses of glutamate receptors and ASICs under different conditions and in the presence of endogenous substances does not allow us to make any bold predictions regarding the exact role of ASICs under different conditions, but it does conceptually explain the involvement of ASICs in synaptic plasticity [38,39,40,41,42] and various CNS disfunctions [43,44,45,46]. Clarification of the role of ASIC in glutamatergic transmission appears to require that both components of transmission be studied together.

## 4. Materials and Methods

All experimental procedures were approved by the Animal Care and Use Committee of the I.M. Sechenov Institute of Evolutionary Physiology and Biochemistry of the Russian Academy of Sciences. Wistar rats (13–19 days old) were anesthetized with sevoflurane and then decapitated. Maximum effort was made to minimize the number of animals used and their suffering. The brains were removed quickly and cooled to 2–4 °C in an ice bath. Transverse slices of the hippocampus, cortex, and striatum (250 μm thick) were prepared using a vibratome (Campden Instruments Ltd., Loughborough, UK) and were stored in a solution containing (in mM) 124 NaCl, 5 KCl, 1.3 CaCl_2_, 2.0 MgCl_2_, 26 NaHCO_3_, 1.24 NaH_2_PO_4_, and 10 D-glucose, aerated with carbogen (95% O_2_, 5% CO_2_). After 2–3 h incubation, the slices were transferred to the recording chamber. Neurons were freed from a slice by vibrodissociation at 50–120 Hz, without any enzymatic treatment of the tissue [47]. Isolated cells were identified based on their morphological and electrophysiological features. 

The whole-cell configuration of the patch-clamp recording technique was used. Patch pipettes (two to five MΩ) were made with a P-97 micropipette puller (Sutter Instruments, Novato, CA, USA). The pipette solution contained (in mM) CsF 100, CsCl 40, NaCl 5, CaCl_2_ 0.5, EGTA 5, and HEPES 10 (the pH was adjusted to 7.2 with CsOH). The series resistance of about 20 MΩ was compensated by 70–80% and monitored during the experiments. The extracellular solution contained (in mM) NaCl 143, KCl 5, CaCl_2_ 2.5, D-glucose 18, MES 10, and HEPES 10 (the pH was adjusted to 7.6 with NaOH). Currents were recorded using an EPC-8 amplifier (HEKA Electronics, Lambrecht, Germany), filtered at 5 kHz, digitized at a sampling rate of 1 kHz using PatchMaster v2x90.2 software (HEKA Electronics, Lambrecht, Germany), and stored on a personal computer. The holding voltage was −80 mV. Drug-containing solutions were made from the extracellular solution and their pH values were adjusted to the required values after the addition of all components. Solutions were applied using the RSC-200 perfusion system (BioLogic Science Instruments, Claix, France) under computer control. The solution exchange time in the whole-cell mode was about 200 ms. To avoid drug accumulation and pH changes in the experimental chamber, the solutions were removed using a suction system. Experiments were performed at room temperature (22–24 °C). Drugs were purchased from Sigma (St Louis, MO, USA), Tocris Bioscience (Bristol, UK), and MedChemExpress (Stockholm, Sweden). 

All experimental data are presented as the mean ± SD estimated from at least five experiments (cells) obtained from at least three animals. Significance of the effects was tested with *t*-tests. Differences were considered significant at *p* < 0.05. pH dependencies were approximated by Hill equation. The design of the experiment and the limited capability of the application system (a maximum of 8 solutions) did not allow an estimation of the entire pH dependence in a single experiment. The data from different cells (n ≥ 5 for each pH value) were pooled together and fitted using the Hill equation for proton concentrations. To pool the results from different cells, the peak amplitude of the response to glutamate receptors agonist (glutamate, kainate, or NMDA) at pH 7.6 in each experiment was assigned a value of 1.0. Approximation error values were taken as the precision measures.

## Data Availability

Original data are available on request.
